# Succession and Replacement of Bacterial Populations in the Caecum of Egg Laying Hens over Their Whole Life

**DOI:** 10.1371/journal.pone.0115142

**Published:** 2014-12-12

**Authors:** Petra Videnska, Karel Sedlar, Maja Lukac, Marcela Faldynova, Lenka Gerzova, Darina Cejkova, Frantisek Sisak, Ivan Rychlik

**Affiliations:** 1 Veterinary Research Institute, Brno, Czech Republic; 2 The University of Technology, Brno, Czech Republic; Leibniz Institute for Natural Products Research and Infection Biology- Hans Knoell Institute, Germany

## Abstract

In this study we characterised the development of caecal microbiota in egg laying hens over their commercial production lifespan, from the day of hatching until 60 weeks of age. Using pyrosequencing of V3/V4 variable regions of 16S rRNA genes for microbiota characterisation, we were able to define 4 different stages of caecal microbiota development. The first stage lasted for the first week of life and was characterised by a high prevalence of *Enterobacteriaceae* (phylum *Proteobacteria*). The second stage lasted from week 2 to week 4 and was characterised by nearly an absolute dominance of *Lachnospiraceae* and *Ruminococcaceae* (both phylum *Firmicutes*). The third stage lasted from month 2 to month 6 and was characterised by the succession of *Firmicutes* at the expense of *Bacteroidetes*. The fourth stage was typical for adult hens in full egg production aged 7 months or more and was characterised by a constant ratio of *Bacteroidetes* and *Firmicutes* formed by equal numbers of the representatives of both phyla.

## Introduction

Colonisation of the intestinal tract of warm blooded animals begins immediately after birth or hatching with parents being the first and most important source of beneficial microbiota. Initial gut colonizers are recruited from facultative anaerobes of the family *Enterobacteriaceae* (phylum *Proteobacteria*). Soon after, representatives of *Firmicutes* begin to appear in chickens within a week after hatching, and lastly, representatives of *Bacteroidetes* appear as part of the intestinal tract microbiota [1]–[6].

The development of gut microbiota in chickens in commercial production is characterized by an absence of contact between newly hatched chicks and adult hens [7]. The initial colonization of the digestive tract in newly hatched chicks is therefore dependent on the microbiota present in the hatchery or the housing environment, and if a pathogen appears in the environment, the poorly populated gut of young chickens may represent an ideal ecological niche for its multiplication. This is why competitive exclusion products containing complex microbiota from healthy adult hens are used for early chicken colonisation and the prevention of infection with pathogens [8]–[11].

The composition of chicken microbiota has been described only for particular age categories [6], [12]–[14]. Furthermore, because of direct economic importance, most of the studies on the development of chicken gut microbiota were performed in broilers. This means that how dynamic or stable the gut microbiota in egg layers is over their entire life, whether it is affected by maturation and onset of laying, or whether it is subjected to changes during extended egg production is completely unknown. In this study, we therefore characterised the development of caecal microbiota in egg laying hens from the day of hatching until 60 weeks of age, when egg production decreased and the flock was slaughtered. Understanding the natural development of gut microbiota may allow for experimental chicken inoculation with specific microbiota and analysis of the effects on growth performance and resistance to pathogen infection. Moreover, identification of mutual interactions between distinct bacterial species may be exploited for inoculating chickens with particular bacteria aimed at reducing colonisation of another species with a zoonotic potential.

## Results

### Development of chicken caecal microbiota

From 292 to 47,657 reads were obtained for 52 individual samples. However, for the majority of samples between 4,000 to 15,000 reads were available that allowed for monitoring the dynamic changes of dominant bacterial taxa forming chicken caecal microbiota. Representatives of 10 different phyla were recorded at least once in the caecal microbiota throughout the hen's life. Out of these, *Proteobacteria*, *Firmicutes* and *Bacteroidetes* formed the vast majority of microbiota across all age categories. Representatives of minority phyla such as *Actinobacteria*, *Deferribacteres, Fusobacteria, Elusimicrobia* and *Synergistetes* formed more than 1% of total microbiota for at least one sampling time whilst representatives of *Tenericutes* and TM7 were only sporadically recorded and never passed above 1% of total microbiota (S1 and S3 Files).

**Figure 1:**
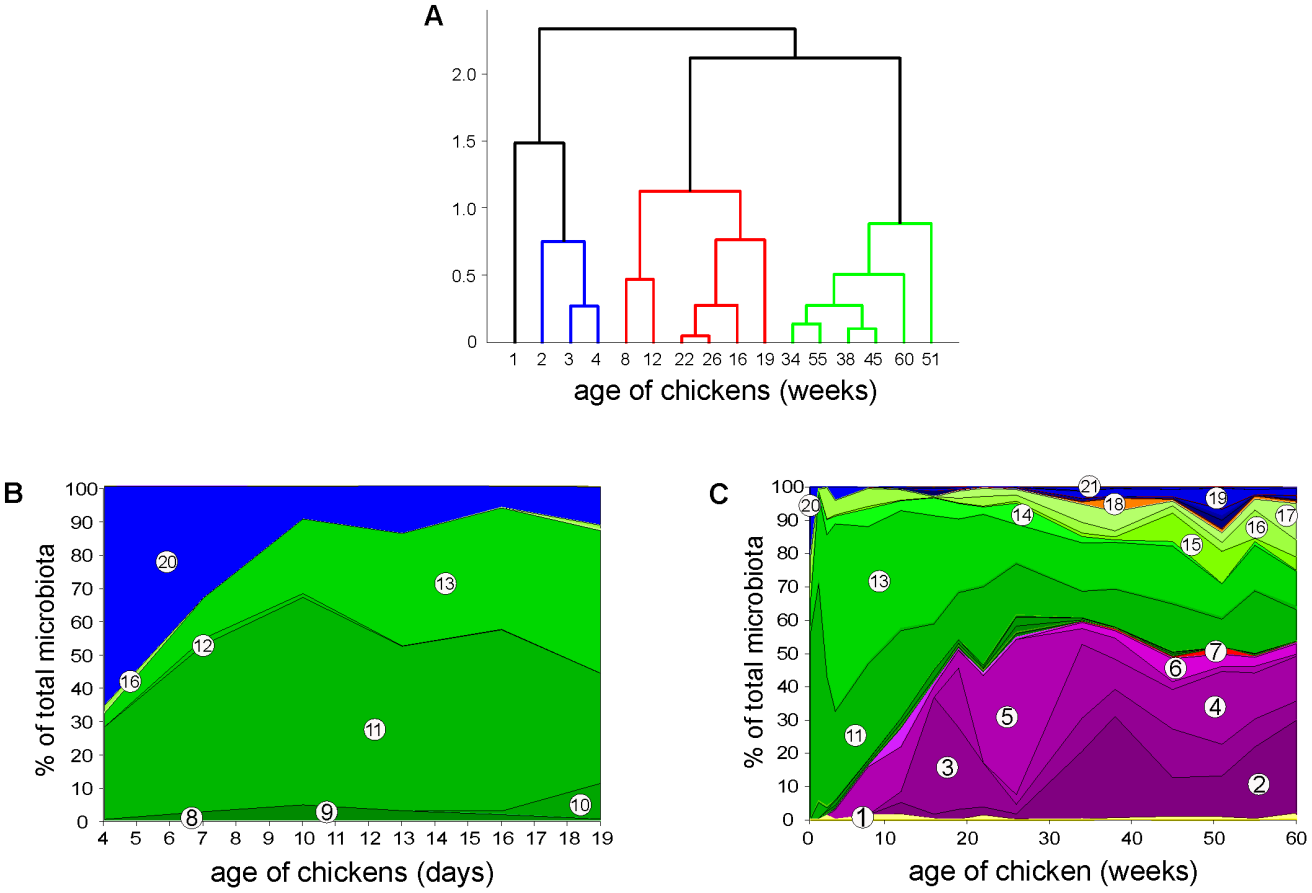
Composition of chicken caecal microbiota as a function of chicken age. Panel A, UPGMA clustering with Mahalanobis distance performed on PCoA data. Panel B, experimental animal house short-term experiment, panel C, long-term on-farm experiment. Green colour, families within *Firmicutes*, violet colour, families within *Bacteroidetes*, blue colour, families within *Proteobacteria*. 1 - *Bifidobacteriaceae*, 2 - *Bacteroidaceae* 3 - *Porphyromonadaceae*, 4 - *Prevotellaceae*, 5 - *Rikenellaceae*, 6 – unclassified *Bacteroidales*, 7 - *Deferribacteraceae*, 8 - *Clostridiaceae* 1, 9 – *Clostridiales* Incertae Sedis XII, 10 - Eubacteriaceae, 11 - *Lachnospiraceae*, 12 - *Peptostreptococcaceae*, 13 - *Ruminococcaceae*, 14 – unclassified *Clostridiales*, 15 - *Veillonellaceae*, 16 - *Lactobacillaceae*, 17 - *Acidaminococcaceae*, 18 - *Fusobacteriaceae*, 19 - *Desulfovibrionaceae*, 20 - *Enterobacteriaceae*, 21 - *Synergistaceae*.

**Figure 2:**
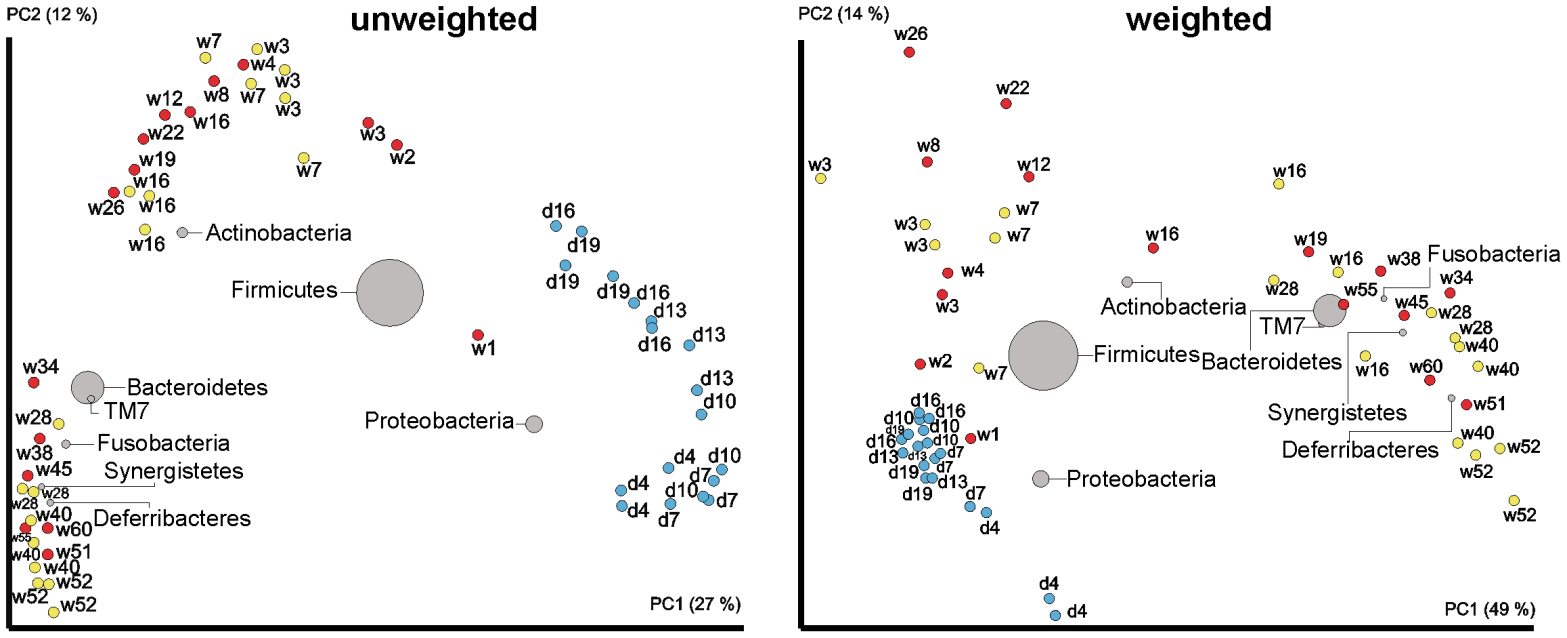
Unweighted and weighted BiPlot PCoA analysis of chicken caecal microbiota. Red spots, pooled samples from the long-term on-farm experiment. Blue spots, individual chicken samples from the short-term animal house experiment. Yellow spots, individual samples from chicken and hens of particular age. “d” stands for age in days, “w” stands for age in weeks. Size and location of the bacterial spots represent their amount and association with microbiota of chickens and hens of a particular age.

**Figure 3:**
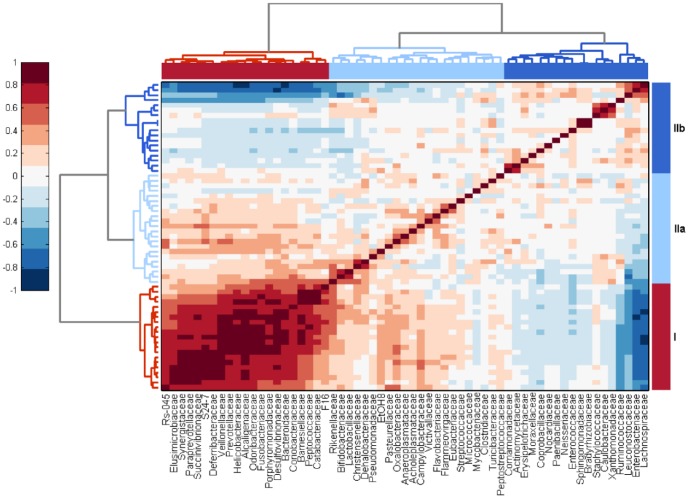
Correlation of bacterial families forming the microbiota in the chicken caecum. Correlation of bacterial families forming the caecal microbiota of chickens and hens is presented as a heat map based on correlation coefficients calculated across all time points and all samples. The correlation coefficients were also used for the calculation of dendrogram trees. Two main clusters, cluster I and cluster II, with 2 subclusters within cluster II could be distinguished. Families within cluster I formed microbiota of mainly adult hens whilst families within cluster IIb were characteristic of young chickens. Dark brown color represents a positive correlation between particular families. Dark blue color represents a negative correlation between particular families.

Four developmental stages of microbiota composition were recorded during the chicken's whole life. This was confirmed by UPGMA clustering performed at family level (Fig. 1), and indirectly supported by PCoA analysis performed at OTU level (Fig. 2) or mutual correlation of individual taxa (Fig. 3). The results from the longitudinal, on-farm study obtained by pyrosequencing on pooled samples were confirmed on individual bird samples by real time PCR specific to 7 selected taxa (File S4). Real time PCR quantification confirmed the trends in microbiota development in time detected by pyrosequencing, however, absolute values of prevalence determined by pyrosequencing and real time PCR for taxons such as *Clostridiales* or *Bacteroidales* were not numerically the same. In addition, the developmental patterns of gut microbiota were confirmed by a short term experiment with chickens up to the age of 19 days and then in an experiment in which we individually tested chickens and hens 3, 7, 16, 28, 40 and 52 weeks of age (S1 and S3 Files). Unweighted PCoA showed that the mere presence of bacterial families at a particular age, i.e. irrespective of their relative representation, was highly uniform. Weighted PCoA plot then showed that there were minor bird to bird variations in relative representation of individual bacterial families (Fig. 2).

The first stage was associated with the first week of the chicken's life and was characterised by a high presence of the representatives from the phylum *Proteobacteria*. These formed nearly 50% of microbiota in the first days of life in the short-term experiment performed in the experimental animal house and 21% of all bacteria of the caecal microbiome in the one-week-old chickens originating from a commercial farm. At the family level, *Proteobacteria* were represented mainly by family *Enterobacteriaceae* and genus *Escherichia* (File S5). The remaining part of caecal microbiota of this age category was formed by representatives of family *Lachnospiraceae* (phylum *Firmicutes*) (Fig. 1B and C).

The second stage of caecal microbiota development was recorded in chickens 2–4 weeks of age reared at the conventional farm. This stage was characterised by a drop in *Proteobacteria* to less than 10% by week 2 of life and by nearly absolute dominance of the representatives of families *Lachnospiraceae* and *Ruminococcaceae* (phylum *Firmicutes*) which formed around 90% of the total microbial population in two-week-old chickens. Representatives of *Lachnospiraceae* (mainly of genera *Blautia* and *Roseburia*) dominated in the caecum in two-week-old chickens and were outcompeted by representatives of *Ruminococcaceae* (mainly of genus *Faealibacterium*) at week 3 of life. Minority families of caecal microbiota belonging to phylum *Firmicutes* included *Lactobacillaceae* (Fig. 1C). A similar caecal microbiota development during the first two stages was confirmed by PCoA also in the chickens kept in the animal house (Fig. 2). The only difference between the commercial farm and clean animal house kept chickens was that the estimated number of different species was approx. 3 times higher in the farm samples than in the animal house samples (compare chao1 indices in S1 and S3 Files which estimate number of bacterial species present in each sample).

The third stage of caecal microbiota development was characteristic for chickens 2-6 months of age. During this stage a gradual succession of the representatives of *Firmicutes* and their replacement with the representatives of *Bacteroidetes* was observed (Fig. 1C and 2). Within phylum *Bacteroidetes*, representatives of *Rikenellaceae* were the first to appear at week 4 followed by the representatives of *Porphyromonadaceae* and *Bacteroidaceae* which were recorded for the first time in 12-week-old chickens. By week 26, i.e. at approx. 6 months of age, representatives of phylum *Bacteroidetes* comprised 55% of the total caecal microbiome of hens (Fig. 1C).

The fourth stage was characteristic of hens older than 7 months. Caecal microbiota of this age category was formed mainly by representatives from *Firmicutes* and *Bacteroidetes*, each of them forming approx. half of the total microbiome. The representatives of *Bacteroidaceae*, though appearing for the first time at week 12, remained at a low level of representation (not exceeding 2% of the total microbiota) until week 34 when they increased to approx. 10% and then fluctuated between 10 and 30% of the total microbiome (Fig. 1C). Within stage 4, *Proteobacteria* re-appeared in the caecal microbiota of hens aged 34 weeks, from which point in time they formed around 5% of the total microbiota. However, genera composition of representatives of *Proteobacteria* in the caecum of hens aged 34 weeks or older and one-week old chickens were different since the representatives of *Proteobacteria* in old hens belonged to genera *Desulfovibrio* and *Succinivibrio* (Fig. 1C and File S5).

Correlation analysis performed at the family level showed that there were 2 main microbiota clusters. Families in cluster I exhibited a high mutual positive correlation (Fig. 3) and were those associated with older birds (compare with Fig. 1C). Within cluster II, two subclusters could be identified. Families within cluster IIa positively correlated with cluster I but not among themselves. Families within cluster IIb were usually those associated with young chickens (compare with Fig. 1C) and were mutually neutral, except for the families *Ruminococcaceae*, *Lachnospiraceae*, *Leuconostocaceae* and *Enterobacteriaceae* which exhibited positive correlations but negatively correlated with bacterial families of cluster I (Fig. 3). Two families comprising potential pathogens localized to cluster I and IIa – *Helicobacteraceae* belonged to cluster I comprising mostly microbiota of old birds and positively correlated with *Veillonellaceae*, *Prevotellaceae* and *Alcaligenaceae*. *Campylobacteraceae* belonged to cluster IIa which was weakly associated with microbiota characteristic for old birds but without any strong link to any other bacterial family.

## Discussion

In this study we were interested in the development of caecal microbiota of egg laying hens kept under the same living conditions throughout their whole life and in the same flock. Such an arrangement minimised confounding factors and allowed us to define 4 stages of microbiota development over the whole life. Immediately after hatching, the caecum became populated by facultative anaerobes of genus *Escherichia,* family *Enterobacteriaceae* (phylum *Proteobacteria*). A week later, the representatives of family *Lachnospiraceae* became predominant, similar to several reports on broiler caecum colonisation [11], [12], [15]–[18]. Similar developmental patterns during the first 3 weeks of life at family or higher-level taxa were observed both for farm-reared chickens and chickens raised in a clean experimental animal house with controlled conditions including air conditioning, air filtration and the absence of contact with rodents or insects (Fig. 1), further confirming our observations. Despite this, it should be reminded that this study was performed in a limited number of chickens collected on just one farm and analysed by just one protocol, i.e. pyrosequencing of 16S V3/V4 variable regions of rRNA gene amplified by PCR. All of these could affect our conclusions which will have to be verified with an extended set of samples and analysed by alternative approaches such as shotgun sequencing or protein mass spectrometry.

The microbial community in the caecum of chickens older than the age of broiler fattening time, i.e. older than 6 weeks, has been characterised much less frequently. Zhao et al. observed around 37% of *Firmicutes* and 10% of *Bacteroidetes* in 8-week-old chickens [19] and Nordentoft et al. characterised microbiota of 18-week-old hens in which the majority of microbiota was formed by the representatives of *Firmicutes* and *Bacteroidetes*, followed by minority populations belonging to phyla *Proteobacteria*, *Actinobacteria* and *Fusobacteria* [14]. In our previous study on the disturbances induced by antibiotic therapy, the microbiota of 15-week-old control chickens differed from that of the 46-week-old chickens with representatives of *Clostridiales* dominating in the faeces of the younger birds [20] and Callaway et al. described the microbiota of 75-week-old hens, two thirds of which were formed by the representatives of *Bacteroidetes* [13]. Finally, we recently characterised faecal microbiota of broilers and hens originating from different European countries and found out that microbiota of broilers, *i.e.* chickens at 3–4 weeks of age, were enriched for *Firmicutes* whilst representatives of *Bacteroidetes* were present mainly in adult hens [21]. Collectively this indicates that conclusions from our longitudinal study are correct though it is clear that the precise timing and/or percentages of representatives of individual phyla will differ from study to study and there might be extensive bird-to-bird variation.

Three core microbiota clusters were defined in humans [22]. Similar to human microbiota, we recorded the existence of *Ruminococcus* enterotype in chickens. However unlike humans, we did not confirm distinct *Prevotella* and *Bacteroides* enterotypes [22]. A likely explanation for the absence of distinct *Prevotella* and *Bacteroides* enterotypes is the fact that chickens and hens were fed uniform feed not allowing for the development of these two enterotypes which seem to be diet dependent [5].

Reasons for the age dependent development of gut microbiota were not investigated in this study experimentally. However, we noticed that the gradual increase of *Bacteroidetes* at the expense of *Firmicutes* (Fig. 1C) correlated with body weight increases during chicken rearing and egg production (File S6). Taking into consideration also the weight of chickens, weekly weight increase represented around 50–80% of body weight during the first weeks of life (File S6). Such a rapid growth requires the efficient functioning of the intestinal tract, both in terms of growth of the intestine itself as well as an increase in nutrient uptake. To cover the energetic demands associated with these processes, butyrate produced by gut microbiota is the most preferred substrate for the respiration of intestinal epithelial cells [23]–[25]. Interestingly, butyrate producers can be found mainly within *Firmicutes* (genera *Faecalibacterium*, *Roseburia* or *Eubacterium*) [26]–[28], and although we did not find representatives of *Eubacterium* in chicken caecal microbiota, the prevalence of *Faecalibacterium* and the whole phylum *Firmicutes* from week 3 of life followed essentially the same profile as body weight increases related to total body weight (File S6). On the other hand, the final products of fermentation by representatives of *Bacteroidetes* include propionate and acetate [29], [30], i.e. the less preferred substrates of colonocytes. In addition, some of the representatives of *Bacteroidetes* are also capable of sulphate release from sulphated chondroitin or mucin produced by host cells [31] which in turn can be respired to H_2_S by *Desulfovibrio* sp. [32] explaining the presence of *Desulfovibrio* only in adult hens. How caecal microbiota can sense the age and body weight of chickens is unknown. It could be affected by the expression of certain proteins or metabolites by the chicken host, e.g. mucin or mucosal IgA, which were reported to be age-dependent and capable of shaping microbiota composition [22], [33], [34]. However, this remains to be experimentally determined.

## Materials and Methods

### Ethical statement

The handling of animals in the study was performed in accordance with current Czech legislation (Animal protection and welfare Act No. 246/1992 Coll. of the Government of the Czech Republic). The specific experiments were approved by the Ethics Committee of the Veterinary Research Institute followed by the Committee for Animal Welfare of the Ministry of Agriculture of the Czech Republic (permit number MZe1479).

### Long-term on-farm development of caecal microbiota

The first monitoring of caecal microbiota development was performed in Lohmann Brown Light chickens (Lohmann Breeders, Germany) in a commercial egg laying hen farm from March 2009 till June 2010. The birds were monitored from week 1 of life when the chicks were transported from a hatchery to the commercial farm until week 60 when the flock was slaughtered (for more information see File S7). As per an interview with the owner, antibiotics were never used during production and the flock was vaccinated against coccidiosis with Livacox T (Biopharm, Czech Republic) on day 10 of life. The diet was changed 6 times during the flock's life (see File S8 for details on feed composition). Three birds were taken from the flock at weeks 1, 2, 3, 4, 8, 12, 16, 19, 22, 26, 34, 38, 45, 51, 55 and 60. The birds were immediately sacrificed and caecal contents were collected and frozen at −20°C until DNA purification.

### Short-term development of caecal microbiota in newly hatched chickens

In the second experiment we verified on-farm observations in a short term experiment with male ISA Brown chicks (Hendrix Genetics, Netherlands) which were obtained from a local commercial hatchery on the day of hatching. The chicks were reared in wire cages in the experimental animal house and allowed ad libitum access to water and pathogen-free feed. Three chicks were sacrificed on day 4, 7, 10, 13, 16 and 19 of life and their caecal contents were collected and frozen at −20°C until DNA purification.

### Verification of the long term experiment

**Figure 4:**
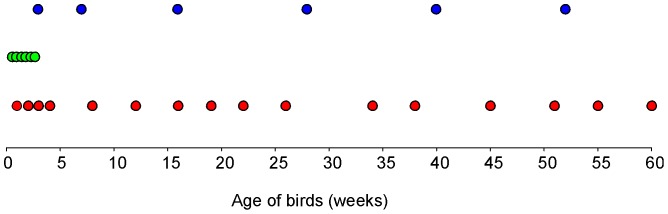
Sampling scheme. Red dots, long-term on-farm development of caecal microbiota during which 3 samples per time point were collected, pooled and pyrosequenced. Green dots, short-term experiment on development of caecal microbiota in newly hatched chickens during which 3 samples per time point were collected and pyrosequenced individually. Blue dots, verification of the long term experiment during which 3 samples per time point were collected and pyrosequenced individually.

In the last experiment targeted at the natural development of gut microbiota of chickens, Lohmann Brown Light chickens or hens aged 3, 7, 16, 28, 40 and 52 weeks were collected from the same egg laying farm as in the first experiment. However, since the hens of different age were kept in different buildings, the hens originated from different flocks. Three birds at each age were sacrificed and caecal contents were collected and frozen at −20°C until DNA purification. The sampling in all 3 experiments is summarised in Fig. 4.

### DNA purification

Caecal samples were homogenised using zirconia silica beads (BioSpec Products) in a MagNALyzer (Roche Diagnostics). Following homogenisation, the DNA was extracted using the QIAamp DNA Stool Mini Kit (Qiagen) according to the manufacturer's instructions and stored at −20°C until use.

### Pyrosequencing and data analysis

Pyrosequencing was performed exactly as described previously [20], [35]. Briefly, the purified DNA was used as a template in PCR with the forward primer 5′ CGTATCGCCTCCCTCGCGCCATCAG – MID-*GGAGGCAGCAGTRRGGAAT* 3′, and reverse primer 5′ CTATGCGCCTTGCCAGCCCGCTCAG- MID- *CTACCRGGGTATCTAATCC* 3′ using HotStarTaq Master Mix Kit following the manufacturer's instructions (Qiagen). The underlined sequences were required for different steps of pyrosequencing while those in italics are sequences complementary to the conserved parts of 16S rRNA genes flanking the V3/V4 hypervariable region. Cycling conditions consisted of a hot start at 95°C for 15 min followed by 30 cycles of incubation at 94°C for 40 s, 55°C for 55 s and 72°C for 60 s. PCR ended with a final extension at 72°C for 5 min. The amplification products were separated electrophoretically in a 1.2% agarose gel, purified using a QIAquick Gel Extraction Kit (Qiagen) and subjected to pyrosequencing. Pyrosequencing was performed using GS Junior Titanium sequencing chemistry and a GS Junior 454 sequencer according to the manufacturer's instructions (Roche). Fasta and qual files generated as an output of the pyrosequencing were uploaded into Qiime software. Quality trimming criteria included no mismatch in MID sequences and a maximum of 1 mismatch in primer sequences. The obtained sequences with qual scores higher than 20 were shortened to the same length of 350 bp and classified with RDP Seqmatch with OTU discrimination set to 97%.

In the long-term on-farm experiment, PCR products originating from 3 individual hens of the same age were pooled prior to sequencing. In the other experiments focusing on the early development of caecal microbiota and microbiota composition of chickens and hens of a particular age, PCR products originating from each chicken were sequenced individually. The raw sequence reads have been deposited in the NCBI Short Read Archive under the accession number SRP046156.

Results from the on-farm longitudinal study were confirmed on the individual chickens by quantitative real time PCR as described previously [36]. Taxon-specific primers were designed from the variable regions of 16S rRNA genes with PRIMROSE software (http://www.cardiff.ac.uk/biosi/research/biosoft/) and the specificity of the primers was verified by the RDP ProbeMatch program. Finally, two primer pairs specific for the conserved regions of 16S rRNA genes (domain *Bacteria* universal primer pairs) served to determine the total bacterial DNA present in these samples (File S9). Real-time PCR was carried out using QuantiTect SYBR Green PCR Kit (Qiagen) and a LightCycler LC480 thermocycler (Roche) with an initial denaturation step at 95°C for 15 min followed by 45 cycles of PCR (94°C for 14 s, 53°C for 30 s and 72°C for 30 s. After PCR, the Ct values of the genes of interest were subtracted from an average Ct value of amplifications performed with the domain *Bacteria* universal primers (ΔCt). The relative amount of each taxon in the total bacterial population was finally calculated as 2^−ΔCt^.

### Statistics

Pyrosequencing data were analysed using Qiime v.1.7.0 software and UniFrac analysis as described previously [20], [35], [37], [38]. Principal coordinate analysis (PCoA) and biplot data visualisation were used to characterise the diversity of the microbial populations tested. UPGMA (Unweighted Pair Group Method with Arithmetic Mean) using Mahalanobis distance performed on PCoA data was used for analysis of the development of microbiota during the chicken's whole life. Bacterial family correlations across all time points and all samples were calculated using Spearman's rank correlation coefficient. Correlations were visualized using cluster heatmap with MATLAB version 2013a (MathWorks).

## Supporting Information

S1 File **Gut microbiota composition in chickens and hens during longitudinal, on-farm monitoring of chicken caecal microbiota development expressed as percentage out of total microbiota.**
(DOC)

S2 File **Gut microbiota composition in chickens during short term monitoring of chicken caecal microbiota development expressed as percentage out of total microbiota.**
(DOC)

S3 File **Gut microbiota composition in chickens or hens 3, 7, 16, 28, 40 and 52 weeks of age expressed as percentage out of total microbiota.**
(DOC)

S4 File **Prevalence of 7 selected bacterial taxons determined in individual chickens or hens during longitudinal, on-farm, determined by real time PCR.**
(PDF)

S5 File **OTU table of all microbiota detected in caecal microbiomes of hens and young chickens classified down to species level.** Full data on all OTUs identified in chickens throughout their whole life are listed in this file.
(XLS)

S6 File **Body weight increases during chicken rearing and the prevalence of butyrate producing bacterium** ***Faecalibacterium*** **sp. in chicken caecal microbiota.**
(PDF)

S7 File **Rearing conditions of the egg laying flock monitored for caecal microbiota development.** This file contains a brief description of conditions under which the flock was reared and kept throughout their whole life.
(DOC)

S8 File **Feed composition used during rearing and egg production.** This file provides additional information on feed composition which was changed 4 times during the whole life of the flock.
(DOC)

S9 File **List of primers used in this study.**
(XLS)
