# Supplementary material for: Succession and Replacement of Bacterial Populations in the Caecum of Egg Laying Hens over Their Whole Life
Source: PLoS One. 2014 Dec 12;9(12):e115142. doi: 10.1371/journal.pone.0115142 (PMC4264878; doi:10.1371/journal.pone.0115142)
Supplement: S1 File — Gut microbiota composition in chickens and hens during longitudinal, on-farm monitoring of chicken caecal microbiota development expressed as percentage out of total microbiota. (DOC) [file pone.0115142.s001.doc]

File S1. Gut microbiota composition in chickens and hens during longitudinal, on-farm monitoring of chicken caecal microbiota development expressed as percentage out of total microbiota. Data are shown from total number of sequences.

|  | Age of chickens or hens (weeks) | | | | | | | | | | | | | | | |
| --- | --- | --- | --- | --- | --- | --- | --- | --- | --- | --- | --- | --- | --- | --- | --- | --- |
| phylum | 1 | 2 | 3 | 4 | 8 | 12 | 16 | 19 | 22 | 26 | 34 | 38 | 45 | 51 | 55 | 60 |
| *Actinobacteria* | 0.13 | 0.13 | 1.42 | 0.15 | 1.46 | 1.79 | 0.43 | 0.21 | 1.50 | 0.29 | 0.32 | 0.68 | 1.05 | 0.90 | 0.46 | 1.94 |
| *Bacteroidetes* | ND | ND | ND | 2.78 | 14.1 | 24.9 | 39.1 | 54.9 | 42.2 | 54.8 | 61.1 | 57.6 | 50.0 | 52.2 | 50.9 | 53.9 |
| *Deferribacteres* | ND | ND | ND | ND | ND | ND | ND | ND | ND | ND | 0.05 | 0.24 | 0.01 | 1.71 | 0.05 | 0.01 |
| *Elusimicrobia* | ND | ND | ND | ND | ND | ND | ND | ND | ND | ND | ND | ND | 0.33 | ND | 0.12 | 0.12 |
| *Firmicutes* | 78.4 | 99.2 | 97.6 | 90.2 | 83.1 | 71.4 | 55.7 | 43.1 | 55.1 | 43.4 | 31.7 | 33.4 | 44.2 | 31.9 | 44.2 | 38.7 |
| *Fusobacteria* | ND | ND | ND | ND | ND | ND | ND | 0.21 | 0.04 | 0.02 | 0.63 | 3.73 | 0.49 | 1.01 | 0.90 | 0.65 |
| *Proteobacteria* | 21.4 | 0.61 | 0.18 | 3.86 | 0.34 | 0.81 | 2.56 | 0.87 | 0.30 | 0.69 | 3.16 | 2.81 | 2.89 | 11.5 | 1.97 | 3.64 |
| *Synergistetes* | ND | ND | ND | ND | ND | ND | ND | ND | ND | ND | 0.77 | 0.34 | 0.59 | 0.14 | 0.53 | 0.15 |
| *Tenericutes* | ND | ND | ND | ND | 0.01 | ND | 0.19 | 0.01 | ND | 0.01 | 0.03 | ND | ND | 0.05 | ND | 0.04 |
| TM7 | ND | ND | ND | ND | ND | 0.36 | 0.54 | 0.09 | 0.13 | 0.36 | 0.31 | 0.05 | 0.09 | 0.08 | 0.17 | 0.06 |
| Number of sequences | 3715 | 7602 | 6755 | 1239 | 15060 | 1138 | 9442 | 14088 | 2330 | 12623 | 6364 | 2167 | 16423 | 47657 | 4187 | 15433 |
| Observed OTUs | 306 | 546 | 894 | 318 | 1311 | 301 | 989 | 1466 | 490 | 1108 | 1050 | 524 | 2470 | 2864 | 960 | 2642 |
| chao1 OTU estimate | 452 | 930 | 1566 | 848 | 2431 | 581 | 2023 | 2846 | 1248 | 1873 | 2248 | 1223 | 4770 | 5769 | 2154 | 6498 |

ND – not detected
