# Supplementary material for: Succession and Replacement of Bacterial Populations in the Caecum of Egg Laying Hens over Their Whole Life
Source: PLoS One. 2014 Dec 12;9(12):e115142. doi: 10.1371/journal.pone.0115142 (PMC4264878; doi:10.1371/journal.pone.0115142)
Supplement: S2 File — Gut microbiota composition in chickens during short term monitoring of chicken caecal microbiota development expressed as percentage out of total microbiota. (DOC) [file pone.0115142.s002.doc]

File S2. Gut microbiota composition in chickens during short term monitoring of chicken caecal microbiota development expressed as percentage out of total microbiota. Data are shown from total number of sequences.

|  | Age of chickens (days) | | | | | |
| --- | --- | --- | --- | --- | --- | --- |
| phylum | 4 | 7 | 10 | 13 | 16 | 19 |
| *Actinobacteria* | ND | 0.10±0.04 | ND | 0.07±0.03 | 0.02±0.00 | 0.03±0.02 |
| *Bacteroidetes* | ND | <0.01 | ND | <0.01 | ND | ND |
| *Firmicutes* | 34.4±17.9 | 66.5±7.31 | 90.3±2.97 | 85.8±6.85 | 93.7±1.46 | 88.6±2.23 |
| *Fusobacteria* | ND | ND | ND | ND | ND | ND |
| *Proteobacteria* | 65.5±17.9 | 33.3±7.30 | 9.72±2.97 | 14.2±6.86 | 6.27±1.46 | 11.4±2.22 |
| TM7 | ND | ND | ND | ND | ND | <0.01 |
| Number of sequences | 2387±316 | 4196±1619 | 3375±1093 | 7623±474 | 16014±2556 | 10757±2054 |
| Observed OTUs | 46±8 | 89±13 | 85±15 | 138±27 | 299±57 | 269±22 |
| chao1 OTU estimate | 59±13 | 153±27 | 116±22 | 194±68 | 539±138 | 446±60 |

ND – not detected
