# Supplementary material for: Succession and Replacement of Bacterial Populations in the Caecum of Egg Laying Hens over Their Whole Life
Source: PLoS One. 2014 Dec 12;9(12):e115142. doi: 10.1371/journal.pone.0115142 (PMC4264878; doi:10.1371/journal.pone.0115142)
Supplement: S3 File — Gut microbiota composition in chickens or hens 3, 7, 16, 28, 40 and 52 weeks of age expressed as percentage out of total microbiota. (DOC) [file pone.0115142.s003.doc]

File S3. Gut microbiota composition in chickens or hens 3, 7, 16, 28, 40 and 52 weeks of age expressed as percentage out of total microbiota. Data are shown from total number of sequences available for each sample and time point.

| Age of chickens (weeks) | | | | | | |
| --- | --- | --- | --- | --- | --- | --- |
|  | 3 | 7 | 16 | 28 | 40 | 52 |
| *Actinobacteria* | 0.3±0.1 | 2.0±1.5 | 0.3±0.2 | 0.1±0.1 | 0.3±0.2 | 0.6±0.1 |
| *Bacteroidetes* | 16.6±16.0 | 10±3.7 | 55.7±4.7 | 52.8±8.6 | 61.2±3.9 | 61.2±5.2 |
| *Deferribacteres* | ND | ND | 0.2±0.3 | 0.1±0.1 | 0.1±0.1 | 0.4±0.1 |
| *Elusimicrobia* | ND | ND | ND | 1.0±0.5 | 0.2±0.1 | 0.6±0.4 |
| *Firmicutes* | 81.9±17.0 | 77.3±7.0 | 40.8±4.1 | 36.6±7.6 | 32.9±3.2 | 31.3±4.8 |
| *Fusobacteria* | ND | ND | 0.8±1.0 | 1.4±1.1 | 0.6±0.3 | 0.1±0.1 |
| *Proteobacteria* | 1.2±1.0 | 10.7±8.0 | 2.0±0.8 | 5.6±0.9 | 3.3±0.9 | 4.8±0.3 |
| *Synergistetes* | ND | ND | ND | 2.0±0.7 | 0.5±0.2 | 0.4±0.2 |
| *Tenericutes* | ND | 0.1±0.1 | ND | ND | ND | ND |
| *TM7* | ND | ND | ND | 0.1±0.1 | 0.3±0.2 | 0.3±0.1 |
| Number of sequences | 1675±1710 | 4698±1975 | 6196±3944 | 4208±1786 | 5785±1146 | 4089±1105 |
| Observed OTUs | 210±160 | 374±124 | 581±398 | 976±321 | 1388±93 | 1088±175 |
| chao1 OTU estimate | 409±328 | 724±255 | 1149±939 | 2522±915 | 3782±171 | 2749±393 |
