# Supplementary material for: Succession and Replacement of Bacterial Populations in the Caecum of Egg Laying Hens over Their Whole Life
Source: PLoS One. 2014 Dec 12;9(12):e115142. doi: 10.1371/journal.pone.0115142 (PMC4264878; doi:10.1371/journal.pone.0115142)

File S4. Comparison of 454 pyrosequencing performed on pooled samples and real time PCR quantification performed individually in 3 samples per time point (shown as average ± standard deviation).

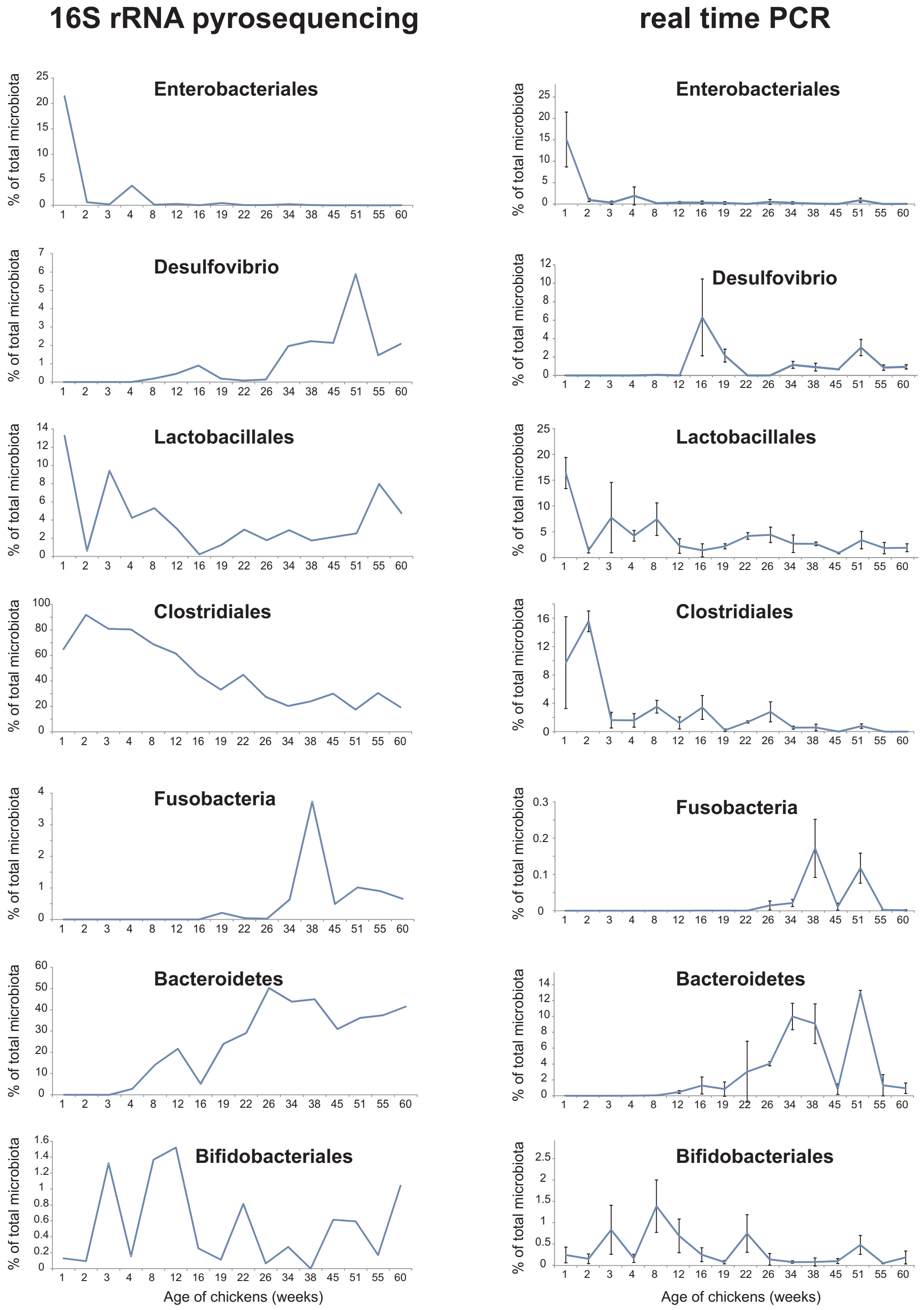

Supplement: S4 File — Prevalence of 7 selected bacterial taxons determined in individual chickens or hens during longitudinal, on-farm, determined by real time PCR. (PDF) [file pone.0115142.s004.pdf]
