# Supplementary material for: Succession and Replacement of Bacterial Populations in the Caecum of Egg Laying Hens over Their Whole Life
Source: PLoS One. 2014 Dec 12;9(12):e115142. doi: 10.1371/journal.pone.0115142 (PMC4264878; doi:10.1371/journal.pone.0115142)

File S6. Body weight increases during chicken rearing and the prevalence of butyrate producing bacterium *Faecalibacterium* sp. in chicken cecal microbiota. Panel A, body weight increase during chicken rearing. Panel B, body weight increases related to the weight of chickens in a given week. Panel C, the prevalence of butyrate-producing *Faecalibacterium* sp. In the cecal microbiome over the whole life of egg laying hens. Red dots indicate time points when feed formula was changed: green dot indicates the onset of egg laying. Since the data from farm owner from 2009 when the samples were collected were no longer available, the body weight increase curves were generated based on the recommendation of Lohmann Breeders which were always strictly followed by the farm owner.

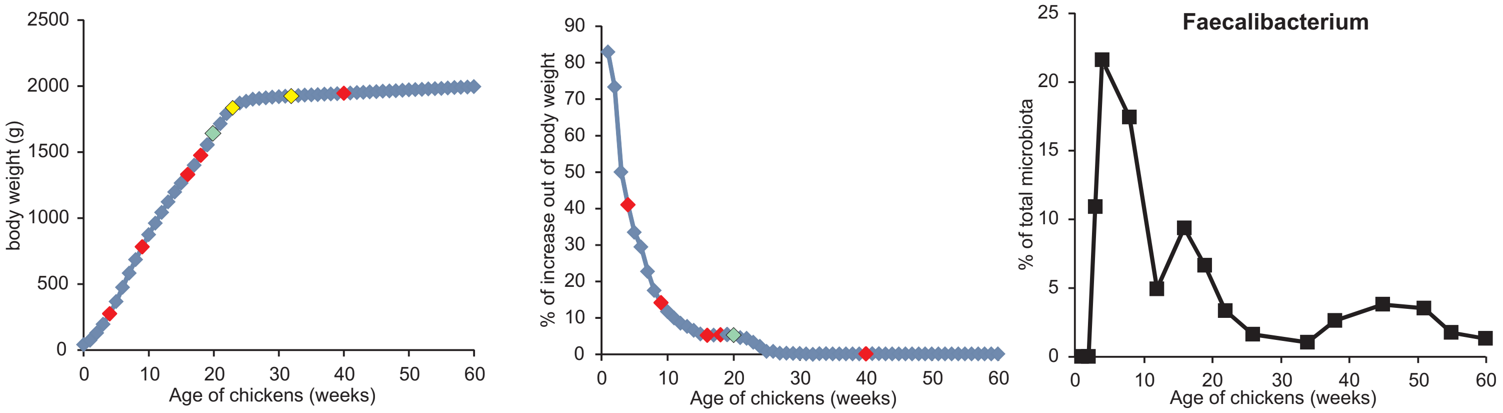

Supplement: S6 File — Body weight increases during chicken rearing and the prevalence of butyrate producing bacterium Faecalibacterium sp. in chicken caecal microbiota. (PDF) [file pone.0115142.s006.pdf]
