# Supplementary material for: Succession and Replacement of Bacterial Populations in the Caecum of Egg Laying Hens over Their Whole Life
Source: PLoS One. 2014 Dec 12;9(12):e115142. doi: 10.1371/journal.pone.0115142 (PMC4264878; doi:10.1371/journal.pone.0115142)
Supplement: S7 File — Rearing conditions of the egg laying flock monitored for caecal microbiota development. This file contains a brief description of conditions under which the flock was reared and kept throughout their whole life. (DOC) [file pone.0115142.s007.doc]

File S7. Rearing conditions of the egg laying flock monitored for caecal microbiota development.

Up to the age of 16 weeks, the chickens were kept in rearing farms. At the age of 16 weeks when the hens reached sexual maturity, the hens were moved to conventional battery cages with controlled temperature, humidity and ventilation. At the same time at week 16, light was extended from 9 to 11 hours per day. During the following weeks the number of light hours per day was continuously increased by 0.5 hour per week until an average day reached 16 hours of light. The peak of egg production was achieved during 25 and 28 weeks of life. After 60 weeks of life production of eggs decreased and the flock was slaughtered.
