# Supplementary material for: Succession and Replacement of Bacterial Populations in the Caecum of Egg Laying Hens over Their Whole Life
Source: PLoS One. 2014 Dec 12;9(12):e115142. doi: 10.1371/journal.pone.0115142 (PMC4264878; doi:10.1371/journal.pone.0115142)
Supplement: S8 File — Feed composition used during rearing and egg production. This file provides additional information on feed composition which was changed 4 times during the whole life of the flock. (DOC) [file pone.0115142.s008.doc]

**File S8.** Feed composition used during rearing and egg production.

| feed mixture |  | K1 | K2 | KZK | N1 | N2 |
| --- | --- | --- | --- | --- | --- | --- |
| week of life |  | 1-3 | 4-8 | 9-18 | 18-40 | 40-60 |
| Lysine | g/kg | 11.5 | 9.8 | 7.6 | 8.2 | 7.6 |
| Methionine | g/kg | 4.8 | 4 | 3.6 | 4.2 | 3.6 |
| Threonine | g/kg | 6.7 | 5.6 | 5.2 | - | - |
| MEd | MJ/kg | 12 | 11.8 | 11.5 | 11.5 | 11.3 |
| Ca | g/kg | 9.2 | 9.1 | 8.8 | 33.5 | 35 |
| P | g/kg | 3.6 | 3.4 | 3.2 | 3.5 | 3 |
| Na | g/kg | 1.5 | 1.5 | 1.5 | 1.5 | 1.5 |
| crude protein | g/kg | 185 | 158 | 135 | 162 | 150 |
| crude fat | g/kg | 33 | 34 | 24 | 25 | 23 |
| fiber | g/kg | 27 | 34 | 49 | 35 | 35 |
| ash | g/kg | 58 | 90 | 58 | 120 | 107 |

Additional information

wheat (60-70%), corn (5-15%), rape seed (5-8%), soybean meal, barely, bran, minerals and vitamins supplementation

Extra mineral supplement Ibemin (Alfamin, Czech Republic) was provided to the flock on week 40.
